# Supplementary material for: Dynamic transcriptomic profiles of zebrafish gills in response to zinc supplementation
Source: BMC Genomics. 2010 Oct 11;11:553. doi: 10.1186/1471-2164-11-553 (PMC3091702; doi:10.1186/1471-2164-11-553)
Supplement: Additional file 2 — Interactive Direct Interaction Network representing the molecular interactions between zinc, copper, iron, calcium and proteins encoded by transcripts changed by zinc supplementation. Mini web-site containing index.html and hyperlinked pages in subdirectory describing a Direct Interaction Network automatically generated based on curated interactions contained within the proprietary PathwayArchitect database. Ovals represent proteins and the circles symbolize metal ions. Objects are coloured by their abundance in zebrafish at the time-point they were significantly different from the control is a scale from -4 fold (dark green) to +4 fold (dark red). Where significant differences were found at more than one time-point, the colour overlay shows expression at the first instance. Dark blue squares denote 'binding', and light blue squares 'expression'; green squares stand for 'regulation', green diamonds for 'metabolism', and green circles for 'promoter binding'. Arrow heads indicate directionality of the interaction where annotated. All nodes and edges can be further interrogated by selecting the relative area of the image. [file 1471-2164-11-553-S2.zip › PathwayArchitect Zn xs DIN/1597798.html]

# BINDING:

|  |  |
| --- | --- |
| Type | BINDING |
| Effect | None |


---

|  |  |
| --- | --- |
| Score | 0 |


---

|  |  |
| --- | --- |
| Reference Count | 17 |


---

|  |  |
| --- | --- |
| Mechanism | Unknown |


---

|  |  |
| --- | --- |
| Reference:0 || SourceID | 244690 |
| Experimental Condition | in-vitro |
| Description | The three dimensional structure of R117h Mutant Rat Anionic Trypsin Complexed with Bovine Pancreatic Trypsin Inhibitor (Bpti) was determined by crystallography. The crystal was grown by using Trypsin II, Bovine Pancreatic Trypsin Inhibitor, pH 8.5 pH 8.5, at 277K. X-ray data were collected using Rotating Anode as a radiation source and were 90.0% complete to a resolution limit of 1.900 angstroms and with an overall Rsym of 8.5%. This crystal contained 2 chains per asymmetric unit and belonged to the space group P 32 2 1. The structure was determined using Molecular Replacement and the modeled structure was refined to an R-factor = 20.8% and had an R-free = 21.0%. PDB: 1CO7, MMDB: 21579. |
| Detection Method | three-dimensional-structure |
| Source | BIND |
  |
|  |  |


---

|  |  |
| --- | --- |
 Reference:1 || SourceID | 223535 |
| Experimental Condition | in-vitro |
| Description | The three dimensional structure of S195a Trypsinogen Complexed with Bovine Pancreatic Trypsin Inhibitor (Bpti) was determined by crystallography. The crystal was grown by using Trypsin, Bovine Pancreatic Trypsin Inhibitor, 24% PEG 4000 0.2 M Liso4 0.1 M Tris pH 8.5 pH 8.0, at 277K. X-ray data were collected using radiation and were 97.3% complete to a resolution limit of 2.2 angstroms and with an overall Rsym of 10.6%. This crystal belonged to the space group P 32 2 1. The structure was determined using Other and the modeled structure was refined to an R-factor = 19.7% and had an R-free = 24.0%. PDB: 3TGJ, MMDB: 8975. |
| Detection Method | three-dimensional-structure |
| Source | BIND |
  ||  |  |


---

|  |  |
| --- | --- |
 Reference:2 || SourceID | 223535 |
| Experimental Condition | in-vitro |
| Description | The three dimensional structure of Rat Anionic N143h, E151h Trypsin Complexed to A86h Ecotin; Zinc-Bound was determined by crystallography. The crystal was grown by using Ecotin, Anionic Trypsin, at 298K. X-ray data were collected using radiation and were 90.9% complete to a resolution limit of 2.2 angstroms with an overall Rsym of 6.5%. This crystal belonged to the space group C 1 2 1. The modeled structure was refined to an R-factor = 17.3% and had an R-free = 26.7%. PDB: 1SLX, MMDB: 4514. |
| Detection Method | three-dimensional-structure |
| Source | BIND |
  ||  |  |


---

|  |  |
| --- | --- |
 Reference:3 || SourceID | 223535 |
| Experimental Condition | in-vitro |
| Description | The three dimensional structure of Rat Anionic N143h, E151h Trypsin Complexed to A86h Ecotin; Nickel-Bound was determined by crystallography. The crystal was grown by using Ecotin, Anionic Trypsin, at 198K. X-ray data were collected using radiation and were 95.5% complete to a resolution limit of 2.0 angstroms with an overall Rsym of 5.4%. This crystal belonged to the space group C 1 2 1. The modeled structure was refined to an R-factor = 17.9% and had an R-free = 25.3%. PDB: 1SLW, MMDB: 4515. |
| Detection Method | three-dimensional-structure |
| Source | BIND |
  ||  |  |


---

|  |  |
| --- | --- |
 Reference:4 || SourceID | 223535 |
| Experimental Condition | in-vitro |
| Description | The three dimensional structure of Rat Anionic N143h, E151h Trypsin Complexed to A86h Ecotin; Copper-Bound was determined by crystallography. The crystal was grown by using Ecotin, Anionic Trypsin, at 298K. X-ray data were collected using radiation and were 96.1% complete to a resolution limit of 2.3 angstroms with an overall Rsym of 6.4%. This crystal belonged to the space group C 1 2 1. The modeled structure was refined to an R-factor = 19.5% and had an R-free = 27.8%. PDB: 1SLV, MMDB: 4516. |
| Detection Method | three-dimensional-structure |
| Source | BIND |
  ||  |  |


---

|  |  |
| --- | --- |
 Reference:5 || SourceID | 223535 |
| Experimental Condition | in-vitro |
| Description | The three dimensional structure of Rat Anionic N143h, E151h Trypsin Complexed to A86h Ecotin was determined by crystallography. The crystal was grown by using Ecotin, Anionic Trypsin, at 298K. X-ray data were collected using radiation and were 98.4% complete to a resolution limit of 1.80 angstroms with an overall Rsym of 4.6%. This crystal belonged to the space group C 1 2 1. The modeled structure was refined to an R-factor = 19.3% and had an R-free = 28.5%. PDB: 1SLU, MMDB: 4517. |
| Detection Method | three-dimensional-structure |
| Source | BIND |
  ||  |  |


---

|  |  |
| --- | --- |
 Reference:6 || SourceID | 223535 |
| Experimental Condition | in-vitro |
| Description | The three dimensional structure of Wild-Type Rat Anionic Trypsin Complexed with Bovine Pancreatic Trypsin Inhibitor (Bpti) was determined by crystallography. The crystal was grown by using Trypsin, Bovine Pancreatic Trypsin Inhibitor, 30% PEG 4000 0.2 M Liso4 0.1 M Tris pH 8.5 pH 8.5, at 277K. X-ray data were collected using radiation and were 88.9% complete to a resolution limit of 1.8 angstroms and with an overall Rsym of 4.9%. This crystal belonged to the space group P 32 2 1. The structure was determined using Other and the modeled structure was refined to an R-factor = 17.7% and had an R-free = 21.0%. PDB: 3TGI, MMDB: 8974. |
| Detection Method | three-dimensional-structure |
| Source | BIND |
  ||  |  |


---

|  |  |
| --- | --- |
 Reference:7 || PMID | 10843853 |
| SourceID | 223535 |
| Species | Rat |
| Experimental Condition | in-vitro |
| Description | The three dimensional structure of Ecotin Mutant M84r, W67a, G68a, Y69a, D70a Bound to Rat Anionic Trypsin II was determined by crystallography. The crystal was grown by using Ecotin, Trypsin II, Anionic, Protein: 15mgML COMPLEX in 10mM Tris pH 8.0 Well: 0.15m Sodium Cacodylate, 0.3m Sodium Acetate, 14% PEG 4K pH 6.5, at 298K. X-ray data were collected using Rotating Anode as a radiation source and were 92.9% complete to a resolution limit of 2.300 angstroms with an overall Rsym of 8.8%. This crystal contained 4 chains per asymmetric unit and belonged to the space group P 1. The structure was determined using the Single Wavelength diffraction protocol and the modeled structure was refined to an R-factor = 18.8% and had an R-free = 23.5%. PDB: 1EZS, MMDB: 13602. |
| Detection Method | three-dimensional-structure |
| Source | BIND |
  ||  |  |


---

|  |  |
| --- | --- |
 Reference:8 || PMID | 10843853 |
| SourceID | 223535 |
| Species | Rat |
| Experimental Condition | in-vitro |
| Description | The three dimensional structure of Ecotin Y69f, D70p Bound to D102n Trypsin was determined by crystallography. The crystal was grown by using Ecotin, Trypsin II, Anionic, Protein: 15mgML in 10mM TRIS pH 8.0 Well: 16.5% PEG 4K, 0.15 M Sodium Cacodylate, 0.3m Sodium Acetate pH 16.5, at 298K. X-ray data were collected using Rotating Anode as a radiation source and were 93.0% complete to a resolution limit of 2.400 angstroms with an overall Rsym of 7.8%. This crystal contained 4 chains per asymmetric unit and belonged to the space group P 1 21 1. The structure was determined using the Single Wavelength diffraction protocol and the modeled structure was refined to an R-factor = 21.8% and had an R-free = 26.5%. PDB: 1EZU, MMDB: 13603. |
| Detection Method | three-dimensional-structure |
| Source | BIND |
  ||  |  |


---

|  |  |
| --- | --- |
 Reference:9 || SourceID | 223535 |
| Experimental Condition | in-vitro |
| Description | The three dimensional structure of Factor Xa Specific Inhibitor in Complex with Rat Trypsin Mutant X99rt was determined by crystallography. The crystal was grown by using Trypsin, 25mM Tris pH7, 10mM CaCl2, 40% Mgso4 pH 7.0, at 287K. X-ray data were collected using Rotaing Anode as a radiation source and were 99.9% complete to a resolution limit of 2.20 angstroms and with an overall Rsym of 10.0%. This crystal contained 1 chain per asymmetric unit and belonged to the space group I 2 3. The structure was determined using Other and the modeled structure was refined to an R-factor = 18.9%. PDB: 1QL9, MMDB: 14072. |
| Detection Method | three-dimensional-structure |
| Source | BIND |
  ||  |  |


---

|  |  |
| --- | --- |
 Reference:10 || PMID | 9819212 |
| SourceID | 223535 |
| Species | Rat |
| Experimental Condition | in-vitro |
| Description | The three dimensional structure of Rat Trypsinogen Mutant Complexed with Bovine Pancreatic Trypsin Inhibitor was determined by crystallography. The crystal was grown by using Trypsin II, Anionic, Pancreatic Trypsin Inhibitor, 22% PEG 3380, 300 mM Ammonium Acetate, 100 mM Sodium Acetate, pH 6.5, at 277K. X-ray data were collected using Rotating Anode as a radiation source and were 93.6% complete to a resolution limit of 1.650 angstroms with an overall Rsym of 5.0%. This crystal contained 2 chains per asymmetric unit and belonged to the space group P 32 2 1. The structure was determined using the Single Wavelength diffraction protocol and the modeled structure was refined to an R-factor = 18.9% and had an R-free = 22.0%. PDB: 1F5R, MMDB: 16558. |
| Detection Method | three-dimensional-structure |
| Source | BIND |
  ||  |  |


---

|  |  |
| --- | --- |
 Reference:11 || PMID | 11420435 |
| SourceID | 223535 |
| Species | Rat |
| Experimental Condition | in-vitro |
| Description | The three dimensional structure of Rat Trypsinogen K15a Complexed with Bovine Pancreatic Trypsin Inhibitor was determined by crystallography. The crystal was grown by using Trypsin II, Anionic, Pancreatic Trypsin Inhibitor, at 277K. X-ray data were collected using Rotating Anode as a radiation source and were 95.7% complete to a resolution limit of 1.550 angstroms with an overall Rsym of 5.6%. This crystal contained 2 chains per asymmetric unit and belonged to the space group P 32 2 1. The structure was determined using the Single Wavelength diffraction protocol and the modeled structure was refined to an R-factor = 19.9% and had an R-free = 20.9%. PDB: 1F7Z, MMDB: 16559. |
| Detection Method | three-dimensional-structure |
| Source | BIND |
  ||  |  |


---

|  |  |
| --- | --- |
 Reference:12 || PMID | 11420435 |
| SourceID | 223535 |
| Species | Rat |
| Experimental Condition | in-vitro |
| Description | The three dimensional structure of Trypsinogen Mutant D194n and Deletion of Ile 16-Val 17 Complexed with Bovine Pancreatic Trypsin Inhibitor (Bpti) was determined by crystallography. The crystal was grown by using Trypsin II, Anionic, Pancreatic Trypsin Inhibitor, 0.2 M Liso4, 0.1 M Tris pH 8.5, at 277K. X-ray data were collected using radiation and were 95.8% complete to a resolution limit of 1.700 angstroms and with an overall Rsym of 5.7%. This crystal contained 2 chains per asymmetric unit and belonged to the space group P 32 2 1. The structure was determined using Other and the modeled structure was refined to an R-factor = 18.4% and had an R-free = 21.8%. PDB: 3TGK, MMDB: 16584. |
| Detection Method | three-dimensional-structure |
| Source | BIND |
  ||  |  |


---

|  |  |
| --- | --- |
 Reference:13 || PMID | 7498454 |
| SourceID | 223535 |
| Experimental Condition | in-vitro |
| Description | The three dimensional structure of Benzamidine in Complex with Rat Trypsin Mutant X99rt was determined by crystallography. The crystal was grown by using Trypsin II, Anionic, pH 7.0 pH 7.0, at 287K. X-ray data were collected using Rotating Anode as a radiation source to a resolution limit of 2.400 angstroms. This crystal contained 1 chain per asymmetric unit and belonged to the space group I 2 3. The structure was determined using the Single Wavelength diffraction protocol and the modeled structure was refined to an R-factor = 19.3%. PDB: 1J14, MMDB: 21364. |
| Detection Method | three-dimensional-structure |
| Source | BIND |
  ||  |  |


---

|  |  |
| --- | --- |
 Reference:14 || PMID | 7498454 |
| SourceID | 223535 |
| Experimental Condition | in-vitro |
| Description | The three dimensional structure of Benzamidine in Complex with Rat Trypsin Mutant X99175190RT was determined by crystallography. The crystal was grown by using Trypsin II, Anionic, pH 7.0 pH 7.0, at 287K. X-ray data were collected using Rotating Anode as a radiation source to a resolution limit of 2.000 angstroms. This crystal contained 1 chain per asymmetric unit and belonged to the space group I 2 3. The structure was determined using the Single Wavelength diffraction protocol and the modeled structure was refined to an R-factor = 17.3%. PDB: 1J15, MMDB: 21365. |
| Detection Method | three-dimensional-structure |
| Source | BIND |
  ||  |  |


---

|  |  |
| --- | --- |
 Reference:15 || PMID | 7498454 |
| SourceID | 223535 |
| Experimental Condition | in-vitro |
| Description | The three dimensional structure of Benzamidine in Complex with Rat Trypsin Mutant X99175190RT was determined by crystallography. The crystal was grown by using Trypsin II, Anionic, pH 7.0 pH 7.0, at 100K. X-ray data were collected using synchrotron radiation to a resolution limit of 1.600 angstroms. This crystal contained 1 chain per asymmetric unit and belonged to the space group I 2 3. The structure was determined using the Single Wavelength diffraction protocol and the modeled structure was refined to an R-factor = 18.9%. PDB: 1J16, MMDB: 21366. |
| Detection Method | three-dimensional-structure |
| Source | BIND |
  ||  |  |


---

|  |  |
| --- | --- |
 Reference:16 || PMID | 7498454 |
| SourceID | 223535 |
| Experimental Condition | in-vitro |
| Description | The three dimensional structure of Factor Xa Specific Inhibitor in Complex with Rat Trypsin Mutant X99175190RT was determined by crystallography. The crystal was grown by using Trypsin II, Anionic, pH 7.0 pH 7.0, at 287K. X-ray data were collected using Rotating Anode as a radiation source to a resolution limit of 1.900 angstroms. This crystal contained 1 chain per asymmetric unit and belonged to the space group I 2 3. The structure was determined using the Single Wavelength diffraction protocol and the modeled structure was refined to an R-factor = 18.3%. PDB: 1J17, MMDB: 21367. |
| Detection Method | three-dimensional-structure |
| Source | BIND |
  |


---

|  |  |
| --- | --- |
